# Effect of noise in processing of visual information

**DOI:** 10.1186/1753-4631-4-S1-S5

**Published:** 2010-06-03

**Authors:** Hiie Hinrikus, Deniss Karai, Jaanus Lass, Anastassia Rodina

**Affiliations:** 1 Department of Biomedical Engineering, Technomedicum, Tallinn University of Technology, Ehitajate Rd 5, 19086 Tallinn, Estonia; 2 Department of Medical Physics, School of Medicine, University of Patras, University Campus, 265 04 Rio Patras, Greece

## Abstract

**Background:**

Information transmission and processing in the nervous system has stochastic nature. Multiple factors contribute to neuronal trial-to-trial variability. Noise and variations are introduced by the processes at the molecular and cellular level (thermal noise, channel current noise, membrane potential variations, biochemical and diffusion noise at synapses etc). The stochastic processes are affected by different physical (temperature, electromagnetic field) and chemical (drugs) factors. The aim of this study was experimental investigation of hypotheses that increase in the noise level in the brain affects processing of visual information. Change in the noise level was introduced by an external factor producing excess noise in the brain.

**Methods:**

An exposure to 450 MHz low-frequency modulated microwave radiation was applied to generate excess noise. Such exposure has been shown to increase diffusion, alter membrane resting potential, gating variables and intracellular Calcium efflux. Nine healthy volunteers passed the experimental protocol at the lower (without microwave) and the higher (with microwave) noise level. Two photos (visual stimuli) of unfamiliar, young male faces were presented to the subjects, one picture after another. The task was to identify later the photos from a group of six photos and to decide in which order they were presented. Each subject had a total of eight sessions at the lower and eight at the higher noise level. Each session consisted of 50 trials; altogether a subject made 800 trials, 400 at the lower and 400 at the higher noise level. Student t-test was applied for statistical evaluation of the results.

**Results:**

Correct recognition of both stimuli in the right order was better at the lower noise level. All the subjects under investigation showed higher numbers of right answers in trials at the lower noise level. Average number of correct answers from *n*=400 trials with microwave exposure was 50.3, without exposure 54.4, difference 7.5%, *p<*0.002. No difference between results at the lower and the higher noise level was revealed in the case of only partly correct or incorrect answers.

**Conclusions:**

Our experimental results showed that introduced excess noise reduced significantly ability of the nervous system in correct processing of visual information.

## Background

Information transmission and processing in the nervous system has stochastic nature. If neurons are driven with identical time-varying stimuli over repeated trials, the timing of the resultant action potentials varies across the trials [1]. This variability is on the order of milliseconds or lower, but because cortical neurons can detect the coincident arrival of action potentials on millisecond timescales, the variability might well be physiologically relevant. A single neuron can respond with different amounts of variability depending on the stimulus conditions and the precision of single-neuron action potential timing on the milli- and sub-millisecond scale has been shown to be behaviourally relevant in perception [1].

Multiple factors contribute to neuronal trial-to-trial variability. Noise and variations are introduced by the processes at the molecular and cellular level (thermal noise, channel current noise, membrane potential variations, biochemical and diffusion noise at synapses) [1]. These different sources of internal noise include changes in the states of neurons and networks, and random processes inside neurons and neuronal networks. Internal noise is generated within the nervous system without external excitation. Total noise in the central nervous system has a complex spatial and temporal structure and affects information processing at macroscopic level (neuronal networks and behaviour). Increase in the noise level can lead to increase of errors in neuronal information processing, change behavioural ability and even affect consciousness [2]. To what extent each of these factors contribute to the total observed trial-to-trial variability remains unclear, especially as stochastic resonance, networking and other effects might reduce variability despite the presence of noise [1,3,4]. Neurons perform highly nonlinear operations that involve high gain amplification and positive feedback. Therefore, small biochemical and electrochemical fluctuations can significantly alter whole-cell responses.

The stochastic processes in nervous system are affected by different physical (temperature, electromagnetic field) and chemical (drugs) factors. Additional excess noise can be generated in the nervous system by an external excitation. The effect of excess noise on cellular or on synapse functions can increase neuronal variability. Thus it becomes possible to compare the amount of variability caused by the internal noise with the variability related to the additional excess noise. The total observed variability gives us an idea of the relative contribution of additional excess noise to trial-to-trial variability.

The aim of this study was experimental investigation of hypothesis that increase in the noise level in the brain caused by an external excitation affects processing of visual information. An exposure to low-frequency modulated microwave radiation was applied for increasing of the noise level. Such exposure has been shown to affect diffusion, membrane resting potential, gating variables and intracellular calcium efflux which obviously contribute to the noise [5-9].

## Methods

### Excess noise

Many stochastic processes are simultaneously working in neurons at the biochemical and biophysical levels. These include protein production and degradation, the opening and closing of ion channels, the fusing of synaptic vesicles and the diffusion and binding of signalling molecules to receptors. According to systematic approach proposed in review [1] the sources of the internal noise are divided into cellular and synapse noise. The cellular noise includes

• Thermal noise (Johnson noise);

• Shot noise (channel current noise, Scottky noise);

• Channels noise (electrical currents produced by the random opening and closing of voltage- or ligand-gated ion channels);

• Variability in resting membrane potential (membrane-potential fluctuations in the absence of synaptic inputs);

• Variability in firing threshold (when the membrane potential is near the firing threshold, the generation of an action potential becomes highly sensitive to noise).

The synapse noise includes

• Spontaneous opening of intracellular Ca^2+^ stores;

• Synaptic Ca^2+^ channel noise;

• Spontaneous triggering of the vesicle-release pathway;

• Spontaneous fusion of a vesicle with the membrane;

• Noise in postsynaptic response (fluctuations in the amplitude of the postsynaptic current);

• Variability in the width (duration of channel opening) of the presynaptic AP;

• Randomness of the diffusion;

• Time variation of the number and density of receptor proteins at synapse; the expression and degradation of proteins is limited by thermodynamic noise.

All these noise sources have a different nature and can be described by different mathematical apparatus. Summing up the effect from large number of the independent noise sources listed above, we can conclude that the total observed noise can be fully accounted as a random process. Fluctuations caused by stochastic nature of the cellular and synaptic processes and instability of the internal states of neurons and networks constitute internal noise of the system N_int_.

The stochastic processes in the nervous system can be affected by different external factors introducing an additional excess noise to the system, N_exc_. The excess noise will then increase neuronal variability. Total neuronal variability *N_total_* consists of internal and excess noise

. 			(1)

Relative part of excess noise can be defined as

. 	 (2)

Presence of the excess noise makes possible to change the total noise level in the nervous system. The excess noise can be added using influence of an external factor to different noise sources listed above.

Modulated microwave radiation is one such external factor that can influence neuronal activity. An electromagnetic disturbance of thermal equilibrium in neurones equivalent to an increase in temperature 1 K, can be introduced by the electric field value of *E* ≈ 1 V/m, and the equivalent field power density 0.0026 W/m^2^[7]. Even a small difference in temperature (2 K) causes changes in transfer rate coefficients of the gating variables and Hodgkin-Huxley model needs correction of the rate constants with the factor 3.48 [10]. Such a disturbance introduced by an external microwave radiation affects two noise sources, the membrane resting potential and the channel noise.

Exposure to microwave has been shown to introduce alterations in intracellular Ca^2+^ efflux [8]. Consequently, the external microwave radiation affects synaptic Ca^2+^ noise sources. 

Electrical forces related to external microwave field cause rotation and displacement of water molecules as electrical dipoles. Therefore influence of microwave radiation on movement of water molecules differs from thermal effect. Microwave radiation has been proven to enhance diffusion about 20% compared to traditional heating [9]. Consequently, the external microwave radiation affects randomness of diffusion in synapses. 

If the modulated at low frequency microwave radiation is applied, alterations in the sensitive to the radiation noise sources are generated at the modulation frequency and, due to nonlinear nature of the interaction, inside the wider low frequency band. This process leads to additional fluctuations in the noise sources and consequently to the excess noise.

### Electromagnetic field exposure

The 450 MHz microwave radiation was generated by the Rhode & Swartz (Munich, Germany) signal generator model SML02. The microwave radiation was 100% pulse-modulated by the pulse modulator SML-B3 (Rhode & Swartz, Munich, Germany), duty cycle 50% at 7 Hz modulation frequency. The signal from generator was amplified by the Dage Corporation (Stamford, CT, USA) power amplifier model MSD-2597601. The generator and amplifier were carefully shielded. The 1W electromagnetic radiation output power was guided by a coaxial lead to the 13cm quarter-wave antenna NMT450 RA3206 by Allgon Mobile Communication AB (Stockholm, Sweden), located 10 cm from the skin on the left side of the head.

The spatial distribution of the electromagnetic radiation power density was measured by the Fieldmeter C.A 43 (Chauvin Arnoux, France) field strength meter. The measurements were performed by the Central Physical Laboratory of the Estonian Health Protection Inspection. During the experiments, the stability of the electromagnetic radiation level was monitored by the IC Engineering (Thousand Oaks, CA, USA) Digi Field C field strength meter. Estimated from the measured calibration curves, the average field power density of the modulated microwave at the skin from the left side of the head was 0.16 mW/cm^2^. This value is lower than recommended by WHO health protection limit for general public based on ICNIRP Guidelines [11].

**Figure 1:**
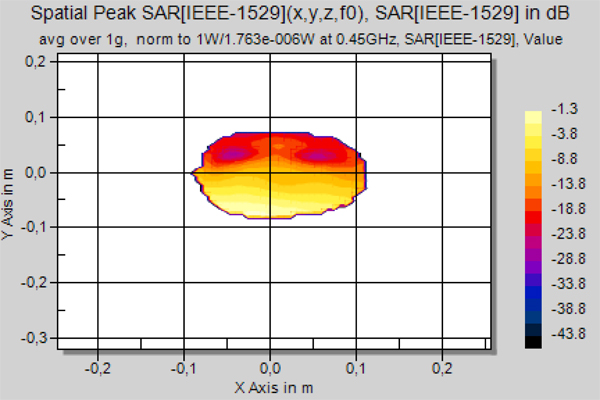
**Calculated SAR in the head model**. Calculated relative distribution of SAR in the cross-section of specific anthropomorphic mannequin (face turned left): 0 dB corresponds to 0.303 W/kg averaged over 1g for 1W antenna input power. The maximum occurs in the muscle near the ear on the left side of the head (lower part in the figure).

The specific absorption rate (SAR) was calculated using SEMCAD (Schmid & Partner Engineering AG, Zurich, Switzerland) software. The finite difference time domain (FDTD) computing method with specific anthropomorphic mannequin specified in IEEE Standard 1528 was applied [12]. Results of calculations are presented in Figure 1. The calculated spatial peak SAR averaged over 1 g was 0.303 W/kg. Relative level of the SAR distribution inside the head varies more than 20 dB.

### Subjects

The experiment was carried out on a group of nine healthy volunteers. The group consisted of four male and five female subjects, aged between 19 and 32 years with a mean age of 22.4 years. All the subjects selected were without any medical or psychiatric disorders and had normal or corrected to normal visual acuity. Subjects were asked to abstain from caffeine and alcohol on the day and a day before the experiment. A questionnaire and a clinical interview were used to evaluate their physical and mental condition (tiredness, sleepiness) before the experiment. The persons who declared tiredness or sleepiness before the experiment were excluded. All subjects passed the experimental protocols at lower (without microwave exposure) and at higher (with microwave exposure) noise level. The subjects were not informed of their exposure during the experiment; however, they were aware of the possibility of being exposed.

The experiments were conducted with understanding and written consent of each participant. The study was conducted in accordance with the Declaration of Helsinki and was formally approved by the Tallinn Medical Research Ethics Committee.

### Experimental procedure

**Figure 2:**
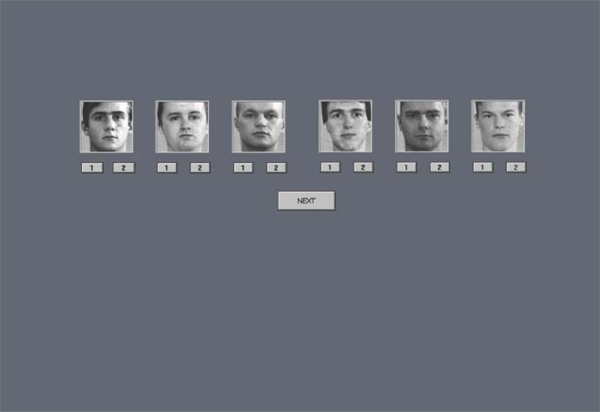
**Visual test as presented in the computer monitor**. An example of the screenshot of the stimulus alternatives for identification of the two successive spatially overlapping test stimuli presented earlier.

The subjects were presented with two successive photos of a male face and the task of a subject was to identify both pictures from a group of six alternative photos and to decide which order they were presented in. Special software was developed in order to present these photos of human faces and to register subject’s ability to identify the images afterwards [13]. Compared to our previous study aimed at face masking effect [13] in this case we investigated the quality of answers (correct-incorrect) to the stimuli. The stimuli were 60 black and white photographs of unfamiliar, young male faces that were presented in the centre of a computer monitor on a gray background. All stimuli were frontal views of faces, which were expressionless and without extraneous cues of identification (glasses, beards, etc.). Two spatially overlapping stimuli were presented in succession. The photos were presented for the durations and with stimulus onset asynchrony specially adjusted for optimum level of performance. Tuning of stimuli presentation and estimation of optimal durations were performed in pilot study before the experiment. In pilot study variable durations of the stimuli and interstimulus interval were applied. The optimal parameters were selected as durations where neither of the two successive visual stimuli would prevail, but each of them could be recognized with compatible, but not 100%, probability. Thus the conditions where only first or second stimuli could have been perceived were excluded. Finally, following durations were selected: first stimulus was presented for 40 ms and the second one for 20 ms with 0 ms interstimulus interval. After each paired presentation, six photos were shown at the centre of the monitor. The task was to match faces that were shown with two of the six alternatives and to decide in which order they were presented. Under each of the six photos there were buttons “1” and “2” which denote order of photos and the responses were made by clicking respective buttons on the computer monitor (Figure 2). Marked wrong order of photos was classified as incorrect answer. The subject was given to respond during 15 seconds. The time between each trial presentation was 25 seconds.

The task was explained and the subjects had 5–10 min for training before the experiment. In the training phase of the test, the participants were asked about their vision and handedness. They had to respond with a mouse using the dominant hand. The participants were sitting in front of the computer monitor with a viewing distance of approximately 50 cm. The experiment was carried out in a dimly lit, sound isolated room. To avoid social stress, subjects were left alone in the room during the experiment. The participants were asked to perform the task calmly and as accurately as they could. They were instructed not to respond if they were not sure about the answer.

The tests were carried out in 16 sessions per subject. Each session consisted of a sequence at the higher (with microwave exposure) and the lower (without microwave exposure) noise level. The random distribution was used for starting the sessions either at the lower or the higher noise level. If the first session started at the higher noise level then next session started at the lower noise level and vice versa. Each subject had a total of eight sessions at the higher and eight sessions at the lower noise level. Each session consisted of 50 trials; altogether a subject made 800 trials, 400 at the higher and 400 at the lower noise level. To avoid subjects becoming aware about condition of the experiment, no any difference in the environment have been present in condition with and without microwave radiation. The antenna was located at 10 cm from the left side of the head and the generator switched on in both experimental conditions, only the radio frequency was switched off.

All subjects performed the same experiment under the same conditions and instructions. The experiment for a subject was carried out during several days over a one-week period. Each subject participated 2–8 sessions during a day. There was at least a 15 min break between the sessions for a subject. During the pauses between the sessions the subject was asked how he/she was feeling, whether he/she felt tired, and if he/she was able to continue the test without discomfort. If the subject could not perform the task, his/her sessions were postponed to another day.

### Data evaluation

The correct and incorrect answers of individual subjects were counted by computer. The mean values of the answers for a group were calculated at the higher and the lower noise level. Relative difference in numbers of right answers at the higher n_h_ and the lower n_l_ noise level was calculated as

.	(3)

Statistical evaluation of results was performed separately for each response class (both correct, one correct, both incorrect, no answer). A two-tailed Student’s t-test with Bonferroni correction for multiple comparisons (number of tests m=4) was performed to evaluate the significance level of comparisons between the results achieved at the higher and the lower noise level for a group and *p*<0.01 was considered as significantly different.

## Results and discussion

Results of experimental test are presented in Table 1. The numbers of responses are divided into four classes separately for the higher and the lower noise conditions: 1) both stimuli recognised correctly and in right order; 2) only the first or the second stimuli recognised correctly and in right order; 3) both stimuli recognised incorrectly or in wrong order and 4) no answer. Calculated for the group statistical parameters differ for the correct (class 1), partly correct (class 2) and incorrect (class 3) answers. There is statistically significant difference (*p*=0.0015) between results at the lower and the higher noise level in the case of correct answers and the number of right answers is higher at the lower noise level, relative difference is 7.5%. 

**Table 1:** Results of the experimental test

Subject	1. Both correct		2. One correct		4. Both incorrect		5. No answer	
	**Higher**	**Lower**	**Higher**	**Lower**	**Higher**	**Lower**	**Higher**	**Lower**

1	38	40	111	117	251	243	0	0
2	60	63	143	114	197	223	0	0
3	62	65	80	71	257	264	1	0
4	62	68	141	149	194	179	3	4
5	9	11	105	110	279	274	7	5
6	35	43	109	119	256	238	0	0
7	54	62	84	77	261	261	1	0
8	86	90	182	187	131	123	1	0
9	47	48	104	105	247	245	2	2
AV	50.33	54.44	117.7	116.6	230	228	1.67	1.22
RD	0.075		-0.009		0.011		0.364	
*p*-value	0.0015		0.79		0.57		0.17	

Numbers of answers for individual subjects at the higher and the lower noise level indicated that 1) both faces were recognised correctly and in right order; 2) the first or the second face was recognised correctly and in right order; 3) both faces were recognised incorrectly or in wrong order and 4) the subject was not able to answer. The average values (AV), relative difference (RD) in average numbers of answers and *p*-values between the results in the higher and the lower noise conditions for a group are presented.

Despite large variability of the numbers of right answers for individual subjects (from 11-9 to 90-86) the numbers are lower in the case of the higher noise level. Regular decrease of the number of correct answers at the higher noise level is evident for all subjects.

In the case of only partly correct or incorrect answers, there are no significant differences between the results at the lower and the higher noise level. Relative difference between numbers of answers in the case of partly correct or incorrect answers is much smaller, only about 1%. Number of subjects who were not able to answer is 36% higher at the higher noise level (last columns in the Table 1), but this difference is not statistically significant.

**Figure 3:**
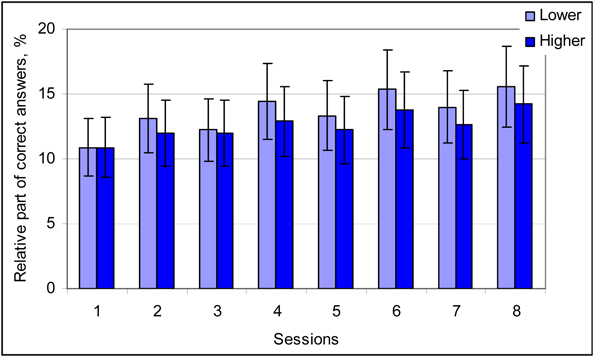
**Results of the test**. Percentage of correct answers in the case of correct recognition of both stimuli in right order for each session at the lower and the higher noise level. Vertical bars denote standard deviation. Number of trials in a session at each noise level n=450; p=0.0017.

The graphs of percentage for correct answers during eight separate sessions at the lower and the higher noise level are presented in Figure 3. Percentage of correct answers in a session varies from 10.9 to 15.6 (average 13.6) at the lower and from 10.9 to 14.2 (average 12.6) at the higher noise level. The variations in relative part of correct answers during different sessions are small. Relative part of right answers is higher at the lower noise level for seven sessions and equal for one session, but the difference between two paired sessions is not statistically significant. Trend of differences between the higher and the lower noise conditions for all sessions leads to statistically significant distinction (p=0.0017). 

Experimental results can be explained taking into account the role of noise in processing of information in the nervous system, where stochastic resonance, neuronal networks and behaviour might reduce effect of noise [1]. In the case of correct answers the internal noise and system behaviour are well balanced and the system is able to make right decision. Any increase in the noise level disturbs this balance and quality of the information processing decreases. Our experimental results show that in this case additional excess noise in the system leads to worse results in correct recognition of stimuli (Table 1, Figure 3).

In the case of partly correct and incorrect answers the system is not able to balance the influence of internal noise and the system is not able to make right decisions even without additional excess noise. In this case additional noise is not a significant factor affecting the decision making process. Our experimental results show that in the case of partly correct or incorrect answers additional excess noise in the system does not affect recognition of stimuli significantly (Table 1). 

It is not possible to measure noise level in the nervous system directly. However, it is likely that the number of errors would increase as noise increases, and therefore the number of correct answers will be higher for the lower noise level. A possible explanation of our results is that the relative difference between correct answers at the lower and the higher noise levels is partly due to the presence of excess noise.

Low value of the relative decrease in the number of correct answers (only 7.5%) can be related to two main reasons. At first, the nervous system networking and stochastic resonance play important role in information processing in the brain and compensate increase in the noise level [1,3]. Therefore real relative alteration in the noise level can be higher than calculated relative difference in numbers of correct answers. At second, the alteration in the noise level caused by modulated microwave is indeed small compared to internal noise in the nervous system. Applied in this study exposure to modulated microwave radiation is proved to affect only part of the internal noise sources; its effect on the other noise sources and total noise level is not clear. Additionally, microwave radiation has inhomogeneous distribution inside the head (Figure 1) and level of the excess noise is lower in some parts of the brain. 

## Conclusions

Our experimental results showed that an additional excess noise introduced to the brain by a low level modulated microwave exposure affects significantly the process of visual perception. The results demonstrated that the excess noise reduced significantly the ability of the nervous system in processing of visual information in the case of correct answers and did not affect the process in the case of partly correct or incorrect answers. Relative decrease of right answers was 7.5% at the microwave field power densities lower than the WHO recommended health protection limits for general public.

## Competing interests

The authors declare that they have no competing interests.

## Authors' contributions

HH conceived of the study, participated in the design of the study and coordination and drafted the manuscript. DK performed SAR distribution modelling. JL participated in the design of the study, performed the statistical analysis and helped to draft the manuscript. AR participated in the design of the study, carried out experimental study and helped to draft the manuscript. All authors read and approved the final manuscript.
